# A novel sophorolipids extraction method by yeast fermentation process for enhanced skin efficacy

**DOI:** 10.1111/srt.13518

**Published:** 2023-11-13

**Authors:** Yae‐rin Lee, Youn Jeong Cha, Sugyeong Jeong, Seok‐Kyun Yun, Younhwa Nho, Seunghyun Kang, Woonha Kim, Juhyun Son, Juyun Kim, Seoyeon Kyung

**Affiliations:** ^1^ COSMAX BTI R&I Center Seongnam South Korea; ^2^ Zero to Seven Inc. Seoul South Korea; ^3^ ZOE BIO BIO‐Material R&I Center Seoul South Korea

**Keywords:** anti‐inflammation, baby skin, bioconversion, *Candida bombicola*, Oji Complex, skin barrier, skin hydration

## Abstract

**Aims:**

Oriental herbs have been used as medicines in the folk remedy for their numerous phytochemicals and bioactivities. In this study, we have selected five Korean traditional medical herbs and applied bio conversion extraction technology, named it as Bioconversion Oji complex, to identify phytochemicals and evaluate skin related efficacies.

**Material and methods:**

The process of two‐step bio conversion was sequentially conducted. The first step of fermentation was to produce biosurfactants using macadamia seed oil with Candida bombicola, and then five natural plants were added to carry out the main fermentation. To evaluate skin improvement efficacy of Bioconversion Oji complex, in vitro and in vivo studies were conducted. We studied HaCaT cells cultured to assess viability, skin anti‐inflammatory, moisturizing and barrier improvement‐related mRNA expression. For efficacy study, 21 participants were tested evaluating anti‐inflammatory, skin moisturizing and skin barrier improving effects of Bioconversion Oji complex compared to Water extraction of Oji (placebo) for the 4 weeks test period.

**Results:**

The application of bioconversion technology highly increased the content of amino acids and lipids within Bioconversion Oji complex, and 23 flavonoids were also identified. Bioconversion Oji complex was found to be non‐toxic and showed significant effects in all parameters tested, including anti‐inflammation, skin moisture, and skin barrier in both in vitro and in clinical studies.

**Conclusions:**

Bioconversion Oji complex has demonstrated skin‐friendly properties with significant beneficial effects on anti‐inflammatory, skin hydration and barrier function properties. This study provides evidence for the use of Bioconversion Oji complex as an active ingredient in cosmetics and skincare products.

## 1 INTRODUCTION

Plants have been utilized as the medicine in the folk remedy in various areas of the world.^1^, ^2^ Over decades, the modern scientific research has provided the reason behind the promising efficacy of such plants introduced in the folk remedy that their diverse phytochemical molecules show positive biological activities in human body.^3^, ^4^ Donguibogam, the Korean traditional medicinal system, was written by Dr Heo Jun in 1613 and it is now considered the ‘bible’ of Oriental herbal medicine due to the historical medical value.^5^ Recently, the natural resources mentioned in skin‐related prescriptions in Donguibogam were analyzed computationally with the skincare function categories. The result demonstrated that the 52 medicinal herbs identified in Donguibogam exhibited definite skin‐related characteristics and 46 of them had been investigated researched to at least one modern scientific study on skincare‐related benefit.^6^ Besides Donguibogam, many other Oriental herbal medicinal studies described the herbs folk remedy for the cure of skin illness benefit and some of them, particularly, focused on their efficacy on baby skin. For instance, a few studies demonstrated the specific herbs complex for baby born in royal family of Joseon dynasty (Korean kingdom for about five centuries, from July 1392 to October. 1897).^7^ Limsanyeojibub is a manual for pregnant women for the birth of a royal baby in Joseon and the *Journal of Hosancheong* is written by Joseon dynasty royal consort Sukbin Choi that described the provisional organization when she gave a birth of the King Yeongjo of Joseon.^8^ Theses manuals mentioned that the extraction of complex of herbs, including *Prunus mume* and *Prunus persica* should be used for the baby's first bathing water to strengthen the skin condition and improve the immune system.^9^ Based on the *Journal of Hosancheong*, the five natural herbs complex, named it as Ojitang, containing *Prunus mume, Prunus persica, Morus alba, Salix alba*, and *Sophora japonica*, was developed.

Bioactive compounds from plants are commonly extracted by organic solvents or hot water. However, these techniques have disadvantages such as the use of solvents with low purity, potential toxicity, low selectivity in extraction, long periods of extraction, and thermal decomposition of heat‐labile compounds.^10^ As an alternative, the extraction technique, called “Bioconversion” has gained so much attention using microbes for their unique features. Biosurfactants, also termed as naturally derived surfactants, are surfactants which are produced by microorganisms and have received wide attention not only for their biodegradability, low toxicity, ecological acceptability but for their skin compatibility and skin effects.^11^ *Candida bombicola* is one of the best‐known examples of microbes naturally producing high amounts of biosurfactants. Multifunctional biosurfactants have several cosmetic applications and especially, sophorolipids have been shown to have good skin compatibility and excellent moisturizing properties.^12^ We have studied emerging extraction techniques called “Oil bioconversion technology” by *Candida bombicola* for their unique metabolic features. *Candida bombicola* is the yeast, able to produce biosurfactants such as glycolipids, lipopeptides, lipoproteins, phospholipids, and fatty acids when grown in a medium composed of two different carbon sources (usually sugar and oil) and a nitrogen source (frequently yeast extract).^13^, ^14^ The components of biosurfactants prior‐mentioned are similar and compatible with those existed in the membrane of skin cell. Their physiochemical properties are also effective for moisturizing dry skin surfaces and improving skin barrier functions.^15^

Since many medicinal materials were used on the baby's skin in the oriental medicinal ancient herbal books, we investigated the characteristics of the baby's skin. Vernix caseosa, the complex of proteolipid metabolic products, functions as barrier to water loss, temperature regulation and keeps innate immunity.^16^ In the previous study, if the vernix caseosa was removed 24 h before the birth of a newborn, moisture loss from the skin and erythema increased.^16^ And in another study, compared to fetal, which has vernix caseosa, the expressions of anti‐microbial peptides are reduced in neonatal, which has no vernix caseosa.^17^ In this respect, we designed the experiment based on assumption that the extraction of Ojitang would protect the baby's skin like vernix caseosa.

In this study, we have selected the five natural herbs, *Prunus mume, Prunus persica, Morus alba, Salix alba*, and *Sophora japonica*, based on the *Journal of Hosancheong*, and named it as Oji complex. Besides, bio conversion extraction technology was applied to extract various phytochemicals from Oji complex for improvement of biological activity of the Oji complex. First, Oji complex containing *Prunus mume, Prunus persica, Morus alba, Salix alba*, and *Sophora japonica*, was prepared. Then, the bio converted macadamia seed oil was prepared using bioconversion process of *Candida bombicola*. After that, Oji complex was added to the bio converted macadamia seed oil and the second bioconversion process were proceeded to extract both hydrophilic and hydrophobic active substances from Oji complex. The second step bio converted extraction of *Prunus mume*, *Prunus persica*, *Morus alba*, *Salix alba*, and *Sophora japonica* was named as “Bioconversion Oji complex”. We not only analyzed the active components (amino acids, lipids, and flavonoids) but also investigated various effects of “Bioconversion Oji complex” in skin compared to Water extraction of Oji complex based on in vitro and in vivo experiments.

## 2 MATERIALS AND METHODS

### 2.1 Materials

*Candida bombicola* UA‐06 (KCTC18592P) strain, Republic of Korea patented microorganism, was used given a deposit number from the Korea Collection for Type Cultures/BRC (KCTC), Korea Research Institute of Bioscience and Biotechnology (KRIBB), Korea. *Macadamia ternifolia* (macadamia) seed oil was purchased from Floratech, a Cargill company (Minneapolis, MN, USA), which was used as the carrier oil during bioconversion extraction process. Water extraction of Oji complex and Bioconversion Oji complex are both composed of following five natural plants. *Prunus mume* and *Prunus persica* (Peach) were purchased from local farm. The mulberry (*Morus alba*), the *Sophora japonica*, and the white willow (*Salix alba*) were purchased at medicinal plants market.

### 2.2 Preparation of plant extract, water extraction of Oji complex

To prepare Water extraction of Oji complex, five raw plants were chopped into small pieces and ground to a fine powder using a blender. The powder (100 g) was extracted for 4 h 30 min with 1000 mL of hot water (120°C, autoclaved). Water extraction of Oji complex was filtered using Advantec filter and concentrated by a vacuum rotary evaporator (Heidolph, Germany).

### 2.3 Preparation of Bioconversion oil, Bioconversion Oji complex

#### 2.3.1 Primary seed culture

*Candida bombicola* UA‐06 (KCTC18592P) strain was inoculated in 150 mL baffled flask containing a 30 mL of seed culture medium and cultured in a shaking incubator (ISS‐7100R, Jeiotech, South Korea) at 25°C, 180 rpm for 24 h. The seed culture medium was composed of 3 g /L yeast extract, 3 g/L malt extract, 5 g/L peptone 10 g/L glucose and 1 g urea. The grown strains were inoculated to a fermentation medium of 10% (v/v).

#### 2.3.2 Preculture

Preculture was inoculated with 10% (v/v) of seed culture and performed under the same conditions as the seed culture, except that 10 g/L of glucose was substituted with 20 g/L of glycerin in the primary medium composition.

#### 2.3.3 Main fermentation

The working volume of main fermentation was 3 L in a 5 L bioreactor (BIOSTAT A plus, Sartorius, Germany). Main fermentation was inoculated with 10% (v/v) of the preculture and incubated at 30°C, 300 rpm under aerobic conditions(10 nL/min) for 96 h.

The main fermentation medium contains 20 g/L glycerin, 25 g/L K_2_HPO_4_, 7 g/L KH_2_PO_4_, 3 g/L NH_4_NO_3_, 2 g/L MgSO_4_ ▪ 7H_2_O, 10 g/L yeast extract and 500 mL/L macadamia oil.

#### 2.3.4 Downstream processing

The downstream process for fermented oil purification consists of centrifugation, sterilization filtration and residual moisture removal. The ferments were centrifuged at 5000 rpm for 15 min, separated into an aqua layer and a lipid layer, and the supernatant (lipid layer) was recovered. The residual moisture of recovered lipid layer was removed using silica gel.

After remove the residual moisture, fermented oil was obtained by sterilization filtration using membrane filter with 0.2 μm pore size.

#### 2.3.5 Preparation of Bioconversion Oji complex using extraction solvent

Extraction solvent was prepared by mixing *Macadamia ternifolia* seed oil and fermented oil. 10.1% of each medicinal plant powder was mixed with 9.9% of extraction solvent and extracted at 40°C for 12 h. Each extract was obtained by filtration using glass fiber filter with 0.45 μm pore size and mixed all together.

### 2.4 Comparative analysis amino acids in water extraction of Oji complex and Bioconversion Oji complex

Bioconversion Oji complex was concentrated 10‐fold by rotary vacuum evaporation at 65°C. The quantitative determination of amino acids was performed at the National Instrumentation Center for Environmental Management (NICEM), Seoul National University (Seoul, South Korea). Primary and secondary amino acids were automatically derivatized into fluorescent substances within the autosampler using o‐phthalaldehyde (Agilent Technologies, USA) and 9‐fluorenyl methyl chloroformate (Agilent Technologies), respectively. Then, the separation of the different amino acid derivatives was performed using an INNO C‐18 column (150 mm × 4.6 mm, 5 μm; Youngjin Biochrom Co., Ltd., South Korea). Standard amino acids were purchased from Agilent Technologies. The mobile phase was a mixture of 10 mM Na_2_HPO_4_ (SigmaAldrich Co.) and 10 mM Na2B4O7 (Sigma‐Aldrich Co.) and water acetonitrile‐methanol (10:45:45 (v/v/v)) (Honeywell Burdick & Jackson Inc., NJ, USA), which was pumped at a constant flow rate of 1.5 mL/min. The quantitative determination of amino acids was performed using a fluorescence detector (excitation: 340 nm; emission: 450 nm). The analysis was performed using the Ultimate 3000 HPLC system (Thermo Fisher Scientific Inc., USA).

### 2.5 Comparative analysis lipids and flavonoids in water extraction of Oji complex and Bioconversion Oji complex

Lipids in Water extraction of Oji complex and Bioconversion Oji complex and flavonoids in Bioconversion Oji complex were additionally identified by HPLC (Ultimate 3000, Thermo Scientific, USA) instrument equipped with a Waters Cortex column and a Triple TOF 5600+ (AB Sciex, USA) instrument at the NICEM, Seoul National University.

### 2.6 Cell culture

Immortalized human keratinocytes (HaCaT) were obtained from American Type Culture Collection (Manassas, VA, USA) and cultured in Dulbecco's modified Eagle medium (Hyclone Laboratories, Inc., Logan, UT, USA) containing 10% fetal bovine serum and 1% antibiotic antimycotic solution at 37°Cin 5% CO_2_. When the cells reached about 80% confluence, cells were subcultured with 4‐(2‐hydroxyethyl)−1‐piperazine ethanesulfonic acid‐buffered saline solution, trypsin/ethylenediaminetetraacetic acid (EDTA) solution.

### 2.7 Cell cytotoxicity assay

The cytotoxicity of water extraction of Oji complex and Bioconversion Oji complex on HaCaT was determined using a 3‐[4, 5‐dimethylthiazol‐2‐yl]−2, 5‐diphenyltetrazolium bromide (MTT) (Sigma Aldrich, St. Louis, MO, USA) assay. Cells were seeded at a density of 2 × 10^4^ cells in 96‐well tissue culture plates. After 24 h, the cells were treated with water extraction of Oji complex and Bioconversion Oji complex concentrations ranging from 1 to 100 μg/mL and then incubated for another 24 h in FBS free media. After removing the medium, cells were treated with 0.5 mg/mL of MTT and incubated for 4 h. After reaction, 100 μL of dimethyl sulfoxide (DMSO) (Signa Aldrich) was added into the cells. Optical density (OD) values were measured at the wavelength of 590 nm using a VersaMax ELISA microplate reader spectrophotometer (Molecular Devices, Silicon Valley, CA, USA)).

### 2.8 RNA extraction and real‐time RT‐PCR analysis

Total RNA was isolated using TRIzol reagent (TaKaRa, shiga, Japan) following the manufacturer's instruction. Approximately 2 μg of total RNA were synthesized to cDNA using Reverse Transcription Premix (Elpis‐biotech, Daejeon, South Korea). Gene expression signals were quantified, and the data were analyzed using the StepOne Plus system software (Applied Biosystems, Foster City, CA, USA). Real‐time RT‐qPCR amplification reactions were performed in a SYBR Green PCR Master Mix with premixed ROX (Applied Biosystems). The following primer pairs (Bioneer, Daejeon, South Korea) were used in the reactions performed in an ABI 7300 following the manufacturer's protocol: β‐actin (F: 5′‐GGCCATCTCTTGCTCGAAGT‐3′ and R: 5′‐GACACCTTCAACACCCCAGC‐3′), TSLP (F: 5′‐ GCTATCTGGTGCCCAGGCTAT −3′ and R: 5′‐ CGACGCCACAATCCTTGTAAT −3′), IL‐1β (F: 5′‐ GTCATTCGCTCCCACATTCT −3′ and R: 5′‐ ACTTCTTGCCCCCTTTGAAT −3′), HAS3 (F: 5′‐ CTTAAGGGTTGCTTGCTTGC −3′ and R: 5′‐ GTTCGTGGGAGATGAAGGAA‐3′), FLG (F: 5′‐ AGTGCACTCAGGGGGCTCACA‐3′ and R: 5′‐ CCGGCTTGGCCGTAATGTGT‐3′), DEFB4 (F: 5′‐ GGTGGTATAGGCGATCCTGTT‐3′ and R: 5′‐ AGGGCAAAAGACTGGATGACA‐3′), CLD1 (F: 5′‐ GCTCTAGAATTCCGAGCGAGTCATGGCCAACGC‐3′ and R: 5′‐ GCTCTAGAATTCTCACACGTAGTCTTTCCCGCT‐3′), and OCLD (F: 5′‐TGTGATGAGCTGGAGGAGGA‐3′ and R: 5′‐ TTCCTGTAGGCCAGTGTCAAA‐3′). The mRNA expression of β‐actin was used as an internal control.

### 2.9 Clinical trial study design

This randomized, double‐blind, placebo‐controlled clinical trial was performed to evaluate and to compare the efficacy of water extraction of Oji complex and Bioconversion Oji complex conducted at the Global Medical Research Center under the approval of the Institutional Review Board (GMRC, IRB no. KDRI‐IRB‐21962, KDRI‐IRB‐21963, KDRI‐IRB‐21964). Twenty‐one volunteers, 20 to 50 s of age, were recruited in this study. This study was conducted for 4 weeks, and all volunteers provided written informed consent.

### 2.10 Sodium lauryl sulfate patch test

Acute irritation induction was conducted by occlusive tests with 1% sodium lauryl sulfate (SLS) to assay the soothing effect of Bioconversion Oji complex. SLS patch testing was performed using 8 mm Finn chamber. For the day before SLS treatment, subject received guidance about the test from the examiner, and filled out basic information, a questionnaire in advance, and a consent form. After washing the forearm with the provided detergent, subjects rest in a constant temperature and humidity condition for 30 min. After partitioning the test site at a point more than 15 cm away from the wrist of one forearm, instrument measurement was performed. 15 μL of 1% SLS is applied as a patch for 24 h using 8 mm finn chamber. After 24 h patch was removed and washed by water. After washing the forearm with the provided detergent again, subjects rest in a constant temperature and humidity condition for 30 min. After dermatologist evaluates the presence or absence of adverse reactions in the test area, test site instrument measurements were performed. Then the test site (Bioconversion of Oji complex treatment), placebo site (Water extraction of Oji complex), and untreated control site were designated. Instructions on precaution, the way how to use the product, and products are provided to subjects. After 2 days, all test, placebo and untreated control sites are washed by water, and after 4 days test site instrument measurements were performed.

### 2.11 Transepidermal water loss

The transepidermal water loss (TEWL) changes before and after using samples in the test area and control area were measured by Tewameter TM‐300 (Corage and Khazaka, Germany). TEWL were measured for irritation calming effect and skin water barrier improvement test. Changes in TEWL were calculated by the following formula; $[\sum_{k\;=\;1}^{n}\;\;\mathrm{kth}\;\mathrm{Measured}\;\mathrm{value}\;\mathrm{immediately}\;\mathrm{after}\;\mathrm{chemical}\;\mathrm{stimulation}\;\mathrm{Measured}\;\mathrm{value}\;\mathrm{after}\;\mathrm{using}\;\mathrm{sample}]/n$

For the skin water barrier improvement test, TEWL were measured before, 2 and 4 weeks after the test, and the moisture barrier improvement rates(%) were calculated as follows; $\{\sum_{k\;=\;1}^{n}\left(\frac{\mathrm{kth}\;\mathrm{pre}-\mathrm{test}\;\mathrm{measurement}-\mathrm{kth}\;\mathrm{measurement}\;\mathrm{after}\;\mathrm{test}}{\mathrm{kth}\;\mathrm{pre}-\mathrm{test}\;\mathrm{measurement}}\times 100\right)\}/n$

### 2.12 Chromameter

The skin redness (a‐value) change was measured using a chromameter CR‐400 (Minoita, Japan) before and after using the sample in the test area and control on the chemical stimulation. Changes in a‐value were calculated by the following formula; $\{\sum_{k\;=\;1}^{n}\;\;\mathrm{kth}\;\mathrm{Measured}\;\mathrm{value}\;\mathrm{immediately}\;\mathrm{after}\;\mathrm{chemical}\;\mathrm{stimulation}\;\mathrm{Measured}\;\mathrm{value}\;\mathrm{after}\;\mathrm{using}\;\mathrm{sample}\}/n$

### 2.13 Corneometer

The improvement of skin moisture content was measured by Corneometer CM 825 (Courage and Khazaka electronic GmbH, Germany). Before, 2 and 4 weeks after the test, the skin moisture contents were measured using a corneometer, and the improvement rate (%) was calculated as follows; ｛$\sum_{k\;=\;1}^{n}\left(\frac{\mathrm{kth}\;\mathrm{pre}-\mathrm{test}\;\mathrm{measurement}-\mathrm{kth}\;\mathrm{measurement}\;\mathrm{after}\;\mathrm{test}}{\mathrm{kth}\;\mathrm{pre}-\mathrm{test}\;\mathrm{measurement}}\times 100\right)$｝/*n*

### 2.14 Statistical analysis

The statistical analyses were performed using two‐tailed Student's *t*‐test. *p*‐Values < 0.05 were considered to be statistically significant. For clinical study, statistical analysis was performed using SPSS statistical package version 25.0. The significances of clinical studies were determined using repeated measures analysis of variance (ANOVA) and independent sample *t*‐test. A *p*‐value of <0.05 was considered statistically significant.

## 3 RESULTS

### 3.1 Quantification of free amino acids in water extraction of Oji complex and Bioconversion Oji complex

Natural moisturizing factor (NMF) is essential for appropriate stratum corneum (SC) hydration, barrier homeostasis, desquamation, and plasticity.^18^ The free amino acids and their derivatives constitute the majority of NMF in the SC.^19^ The amino acid profiles of Water extraction of Oji complex and Bioconversion Oji complex were evaluated using HPLC. The OPA amino acid derivation method allowed identifying and quantifying amino acids in each sample, whereas HPLC amino acid analysis resulted in reliable detection and high peak resolution. Table 1 summarizes the kinds and dose of amino acids detected in the Water extraction of Oji complex and Bioconversion Oji complex 10× (10‐fold concentrated sample of Bioconversion Oji complex). With the great advantages of bioconversion technology, the number of amino acids was increased from sixteen to nineteen, and the total concentration of amino acids showed a 6.5‐fold increase.

**TABLE 1:** Quantitative amino acids analysis (mg/L, ppm) between water extraction of Oji complex and Bioconversion Oji complex 10×.

No.	Amino acids	Water extraction of Oji complex	Bioconversion Oji complex 10×
**1**	Aspartic acid	0.24	10.14
**2**	Glutamic acid	0.20	51.39
**3**	Asparagine	1.79	67.18
**4**	Serine	0.08	8.64
**5**	Glutamine	ND	14.32
**6**	Histidine	ND	3.42
**7**	Glycine	0.05	2.06
**8**	Threonine	0.06	13.04
**9**	Arginine	5.88	13.63
**10**	Alanine	0.13	67.10
**11**	GABA	0.27	105.71
**12**	Tyrosine	0.04	24.78
**13**	Valine	0.06	35.98
**14**	Tryptophan	ND	24.92
**15**	Phenylalanine	0.03	17.99
**16**	Isoleucine	0.02	19.72
**17**	Leucine	0.05	20.14
**18**	Lysine	0.11	2.86
**19**	Hydroxy proline	ND	ND
**20**	Proline	0.26	98.10
	Total	9.25	601.12

*Note*: Bioconversion Oji complex 10×: 10‐fold concentrated sample of Bioconversion Oji complex.

Abbreviation: ND, not detectable.

### 3.2 Qualification analysis of lipids in water extraction of Oji complex and Bioconversion Oji complex

Lipids are one of the fundamental components of the skin. Glycerolipids, sphingolipids, and sterols are the three major classes of membrane lipids^20^ and both phospholipids and glycolipids are polar lipids which are important constituents of natural cell membranes.^21^

Sophorolipids are extracellular glycolipids produced mainly by Candida species when grown on carbohydrates or fatty acids or their mixtures.^22^ After analyzing with TOF/LC/MS, lipids components in Water extraction of Oji complex and Bioconversion Oji complex were searched through the lipid library program. As a result of comparative analysis of lipids between Water extraction of Oji complex and Bioconversion Oji complex, 23 derived lipids were newly identified after applying these bioconversion processes which constituted skin barrier and regulate the permeability barrier homeostasis. The list of compound lipids are listed in Table 2.

**TABLE 2:** Qualitative lipids analysis in Bioconversion Oji complex.

No.	Compound lipids	Water extraction of Oji complex	Bioconversion Oji complex
**1**	Hydroxy fatty acids	ND	19‐hydroxy‐nonadecanoic acid
			24‐hydroxy‐10Z‐tetracosenoic acid
			25‐O‐(2′'‐beta‐D‐glucopyranosyl‐D‐glucopyranosyl)−25‐ hydroxy‐11E‐eicosenoic acid
**2**	Sphingolipids		Dihydrosphingosine
			SM(d16:1/18:1)
			Sphingosine
			Glycosylceramide
**3**	Phospholipids		PE 17:0/18:1
			PC
			PE‐cer(d14:1(4E)/20:0)
			PG
			PI
**4**	Aminolipids		N‐oleoyl GABA
			N‐stearoyl serine
**5**	Fatty acids		Eicosenoic acid
			Ricinoleic acid
			Traumatic acid
			Palmitic acid
			Oleic acid
**6**	Glycerolipids		1‐oleylglycerol
			1,2‐dioctanoyl‐sn‐glycerol
			1‐palmitoylglycerol
			2‐Linoleoylglycerol

Abbreviation: ND, not detectable.

### 3.3 Qualification analysis of flavonoids in Bioconversion Oji complex

Natural flavonoids have the potential photoprotection property because of their UV absorption, their ability to act as direct and indirect antioxidants as well as anti‐inflammatory and immunomodulatory agents.^23^ In addition to analyzing free amino acids and lipids, we additionally conducted the qualification analysis of flavonoids in the Bioconversion Oji complex. After analyzing with TOF/LC/MS, flavonoids components were searched through the library program. As a result of flavonoids analysis of the Bioconversion Oji complex, 23 flavonoids were identified and listed in Table 3.

**TABLE 3:** Qualitative flavonoids analysis in Bioconversion Oji complex.

No.	Compound flavonoids	Bioconversion Oji complex
**1**	Flavonols & flavonol Glucosides	Quercetin 3‐O‐malonyglucoside
**2**		Luteolin −6‐ glucoside
**3**		Trifolin
**4**		Kaempferol
**5**		Prunin
**6**		Herbacetin 3,8‐diglucoside
**7**		quercetin‐3‐O‐deoxyhexosyl(1‐2)pentoside
**8**		Quercetin 3‐O‐glucoside
**9**		Spiraeoside
**10**		Rutin
**11**		Quercetin‐3‐O‐rutinoside
**12**		Dihydrokaempferol
**13**		Hirsutrin (Isoquercitrin)
**14**		Quercetin
**15**	Flavones and flavones glucosides	Calendoflavoside (Isorhamnetin 3‐neohesperidoside)
**16**		Luteolin −3′,7‐di‐O‐glucoside
**17**	Flavanones and flavanones glucosides	Naringenin
**18**		Naringenin‐7‐O‐glucoside
**19**		Miscanthoside
**20**	Flavanols	Catechin
**21**	Anthocyanins and anthocyanin glucosides	Procyanidin
**22**		Keracyanin
**23**		Delphinndin‐3‐rutinoside

### 3.4 Effects of Bioconversion Oji complex on HaCaT cytotoxicity

**FIGURE 1:**
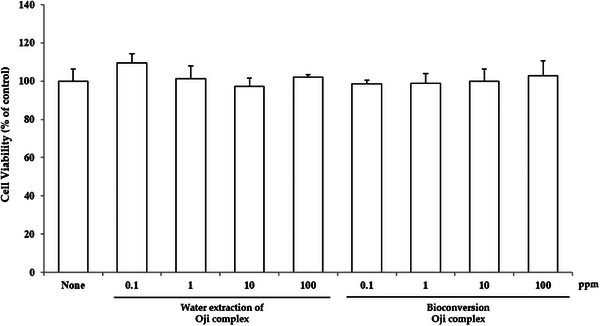
The effects of water extraction of Oji complex and Bioconversion Oji complex on cell proliferation in HaCaTs. HaCaTs were treated with indicated concentrations of water extraction of Oji complex and Bioconversion Oji complex for 24 h. Cell proliferation was measured by the 3‐[4, 5‐dimethylthiazol‐2‐yl]−2, 5‐diphenyltetrazolium bromide (MTT) assay. Results are expressed as mean values ± SE of three independent experiments. ^**^ *p* < 0.01, ^*^ *p* < 0.05 compared to the control.

To determine whether Water extraction of Oji complex and Bioconversion Oji complex could induce cell cytotoxicity, MTT assay was performed on HaCaT. Cultured HaCaTs were incubated with Water extraction of Oji complex and Bioconversion Oji complex with various concentrations ranging from 0.1 to 100 ppm for 24 h. As shown in Figure 1, neither group showed a decrease in cell proliferation levels. These results suggested that both Water extraction of Oji complex and Bioconversion Oji complex may have no cytotoxicity in HaCaT with exposure concentration.

### 3.5 Anti‐inflammatory effects of Bioconversion Oji complex

**FIGURE 2:**
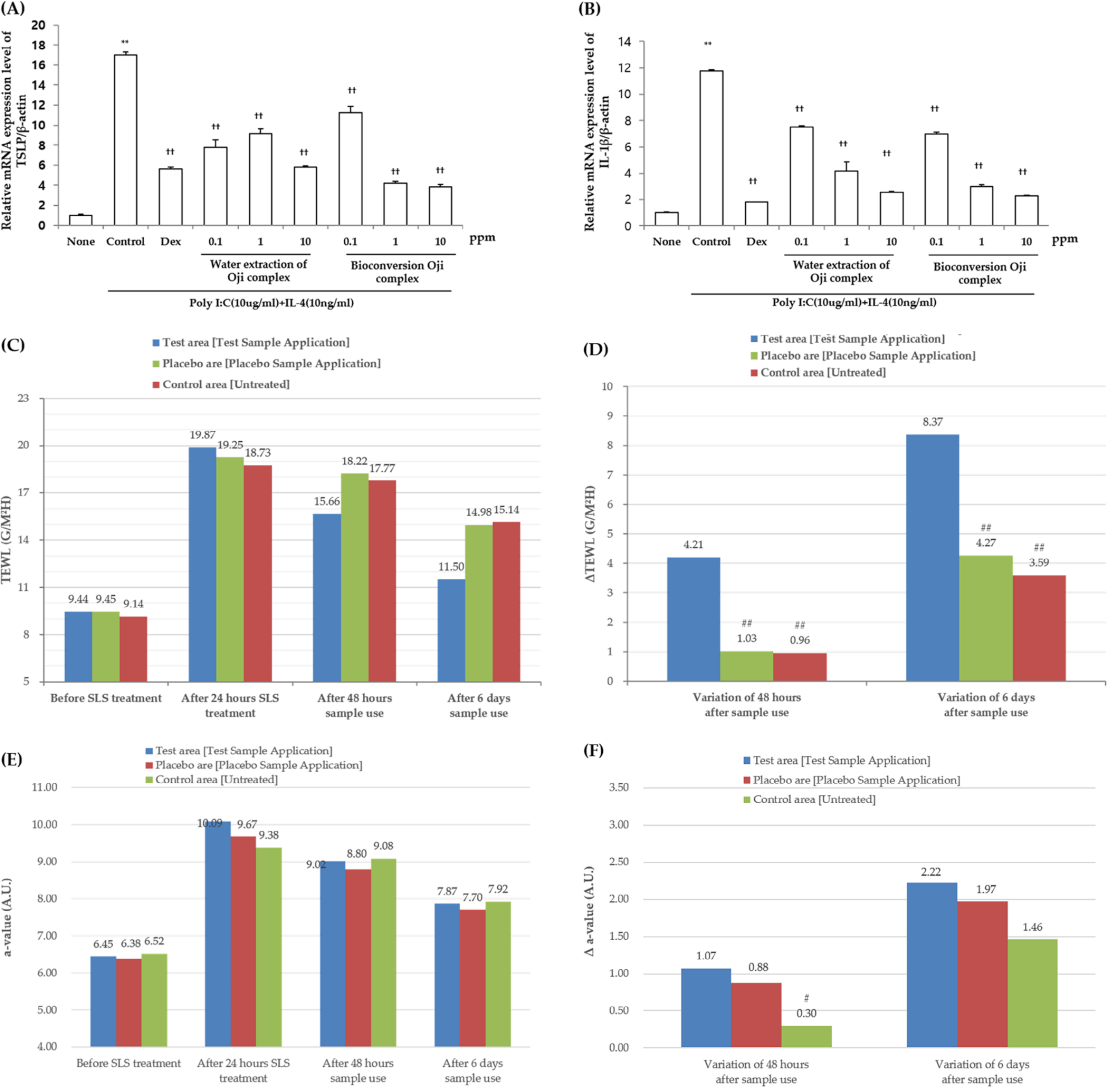
Skin calming effects of Bioconversion Oji complex (Test sample) and water extraction of Oji complex (Placebo sample) on human skin in vitro and in vivo. The HaCaTs were treated with water extraction of Oji complex and Bioconversion Oji complex at the indicated doses for 24 h, and the relative mRNA levels of (a) TSLP and (b) IL‐1β were measured by qRT‐PCR. The means ± SEs are the average of three independent experiments. ^**^ *p* < 0.01, ^*^ *p* < 0.05 compared to the non‐treated group, and ^††^ *p* < 0.01, ^†^ < 0.05 compared to the control group. In clinical study, (C) TEWL, (D) Variation of TEWL were measured and calculated by Tewameter TM300 instrument. (E) a‐value and (F) Δa‐value were measured and calculated by Chromameter CR‐400. Data (*n* = 21) represent mean. #*p* < 0.05 indicates a statistically significant differences compared to control. TEWL, transepidermal water loss.

It has been reported that Thymic stromal lymphopoetin (TSLP) is a cytokine overexpressed in response to allergens and links innate or acquired immunity.^24^ Also, TSLP directly stimulates nerve neurons to feel itching and induces scratching behavior to induce additional inflammation. In particular, TSLP has been suggested as an allergen in atopic patients.^25^ When normal keratinocytes are cultured under IL‐4 10 ng/ml and Poly I:C 10 μg/ml culture conditions, TSLP induction was reported in previous study, so we used that culture condition in HaCaT cell line.^26^ It is also known that interleukin 1β (IL‐1β) induced TSLP secretion,^27^ and it mediates acute immunity in the skin and causes inflammatory responses,^28^ we considered IL‐1β as main inflammatory cytokine marker. Here, we quantified the mRNA expression levels of these inflammatory cytokines, such as TSLP as well as IL‐1β, in the presence or absence of Water extraction of Oji complex and Bioconversion Oji complex for 24 h using RT‐qPCR. Since Dexamethasone has been commercially used as a glucocortinoid‐based atopic treatment and has been reported to reduce TSLP in normal keratinocyte,^29^ Dexamethasone was used as a positive control. The results showed that the treatment of Bioconversion Oji complex in HaCaTs significantly decreased mRNA expression levels of IL‐1β in a dose dependent manner (Figure 2B). And the mRNA expression levels of TSLP were also decreased with Bioconversion Oji complex in a dose dependent manner (Figure 2A). Therefore, it is reasonable to speculate that the Bioconversion Oji complex can sooth damaged skin by alleviating the inflammatory cytokine mRNA expression of HaCaTs. Thus, we next conducted a clinical trial to see if Bioconversion Oji complex relieves skin irritation. As a result of testing on 21 subjects for the 7 days test period, the skin anti‐inflammatory effect of Bioconversion Oji complex sample was evaluated. Bioconversion Oji complex was applied to the test area and Water extraction of Oji complex was applied to the placebo area where TEWL and erythema (a‐value) were increased by damaging the skin barrier through SLS irritation. The increased TEWL showed statistically significant (*p* < 0.05) decrease in test group, being 4.21 g/m^2^h and 8.37 g/m^2^h respectively, after 48 h and 6 days of sample application compared by slight decrease in TEWL in placebo area and control area (Figure 2C,D). Also, after 48 h of sample use, it was confirmed that the erythema (a‐value) of the skin decreased to a statistically significant level (*p* < 0.05) compared to the control area and decreased numerically compared to the placebo area (Figure 2E,F).

### 3.6 Skin moisturizing effects of Bioconversion Oji complex

**FIGURE 3:**
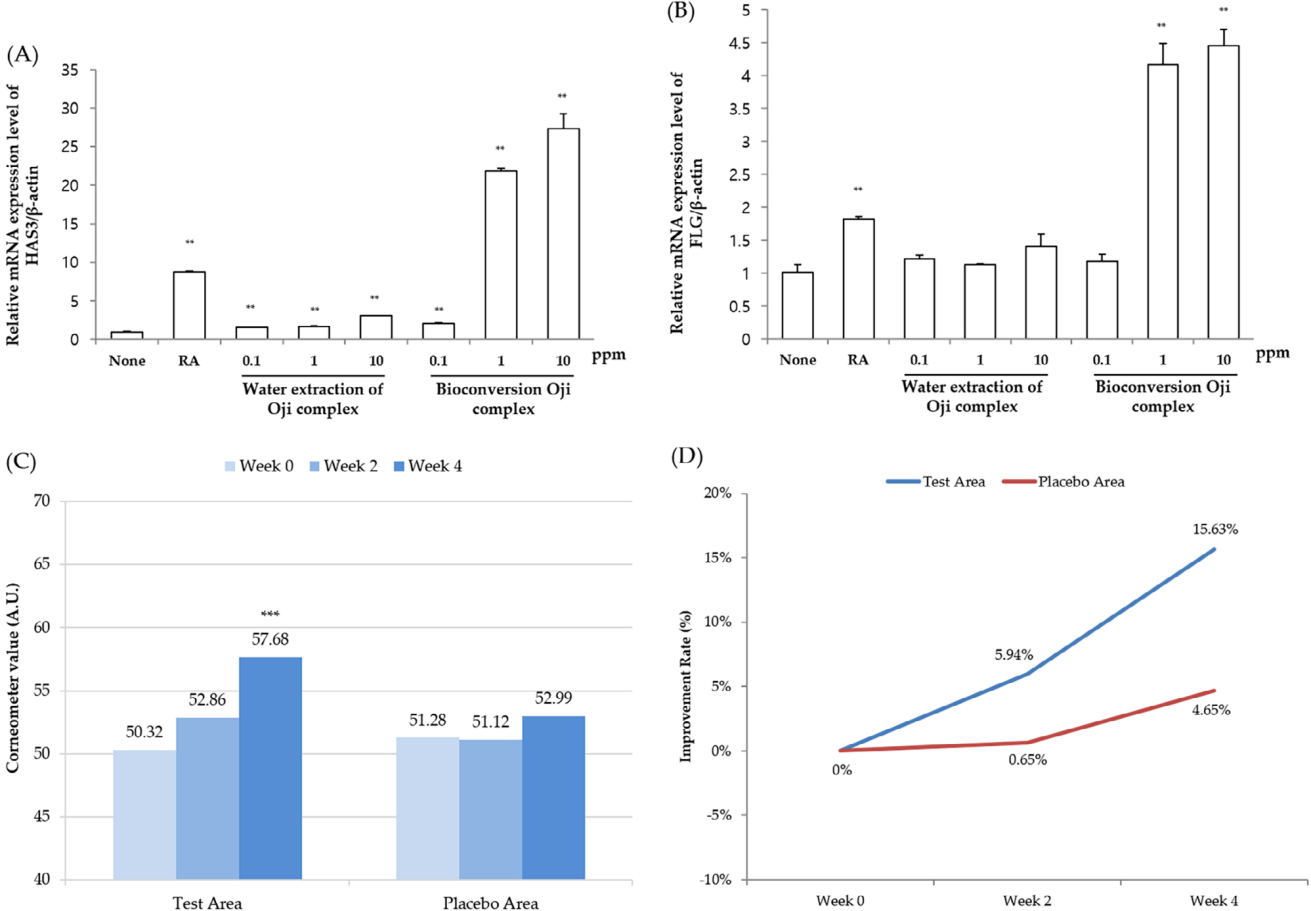
Skin moisturizing effects of Bioconversion Oji complex (Test sample) and water extraction of Oji complex (Placebo sample) on human skin in vitro and in vivo. The HaCaTs were treated with Bioconversion Oji complex or water extraction of Oji complex at the indicated doses for 24 h, and the relative mRNA levels of (a) HAS3 and (b) FLG were measured by qRT‐PCR. The means ± SEs are the average of three independent experiments. ^**^ *p* < 0.01, ^*^ *p* < 0.05 compared to the non‐treated group. In clinical study, (C) Changes of the skin water content and (D) improvement rate of skin water content was measured and calculated by Corneometer CM825 instrument. Data (*n* = 21) represent mean. ^***^ *p* < 0.001 indicated a statistically significant differences compared to non‐treated control, ^##^ *p* < 0.01, ^###^ *p* < 0.001 indicates a statistically significant differences compared to placebo control.

Hyaluronan (Hyaluronic acid, HA) is a structural protein found in the epidermis and dermis of the skin, having the property of holding a large amount of water even at low concentrations and synthesized by hyaluronan synthase (HAS) in vivo.^30^ It is known that there are three types of HAS, among which HAS3 is present in the epidermis and is known to synthesize hyaluronan.^31^ Increased HAS3 gene expression is induced by keratinocyte growth factor, which activates the migration of epidermal cells, promotes medium‐sized HA production in epidermal cells, and helps wound regeneration.^32^ Filaggrin is a structural protein found in the SC and is produced by decomposition of profilagrin in the epidermal layer.^33^ Profilagrin is regulated by the FLG gene,^34^ and filaggrin monomer decomposed from profilaggrin acts as a NMF in the skin and plays an important role in skin moisturizing.^35^, ^36^ HAS3 and Profilaggrin are also expressed in HaCaT used in this experiment.^37^, ^38^ and retinoic acid (RA), which is well known to induce the accumulation of hyaluronate (a hyaluronan analog) in the human epidermis,^39^ has been used as positive control to increase HAS3 and FLG gene expression in HaCaTs.^40^ The results showed that the treatment of Bioconversion Oji complex in HaCaTs significantly increased mRNA expression levels of HAS3 and FLG in a dose dependent manner (Figure 3A,B). Thus, we next conducted a clinical trial to see if Bioconversion Oji complex has skin moisturizing improvement effect. As a result of testing on 21 subjects for the 4 weeks test period, the skin moisturizing effect of Bioconversion Oji complex sample was evaluated. Bioconversion Oji complex was applied to the test area and Water extraction of Oji Complex was applied to the placebo area. The test area using the Bioconversion Oji complex sample showed a statistically significant (*p* < 0.05) increase in the skin water content after 4 weeks from the sample application compared to before sample application. Also, a statistically significant difference (*p* < 0.05), 3.3‐fold increase in skin water content improvement, was shown in comparison with the placebo area with Water extraction of Oji complex (Figure 3C,D)

### 3.7 Skin barrier improving effects of Bioconversion Oji complex

**FIGURE 4:**
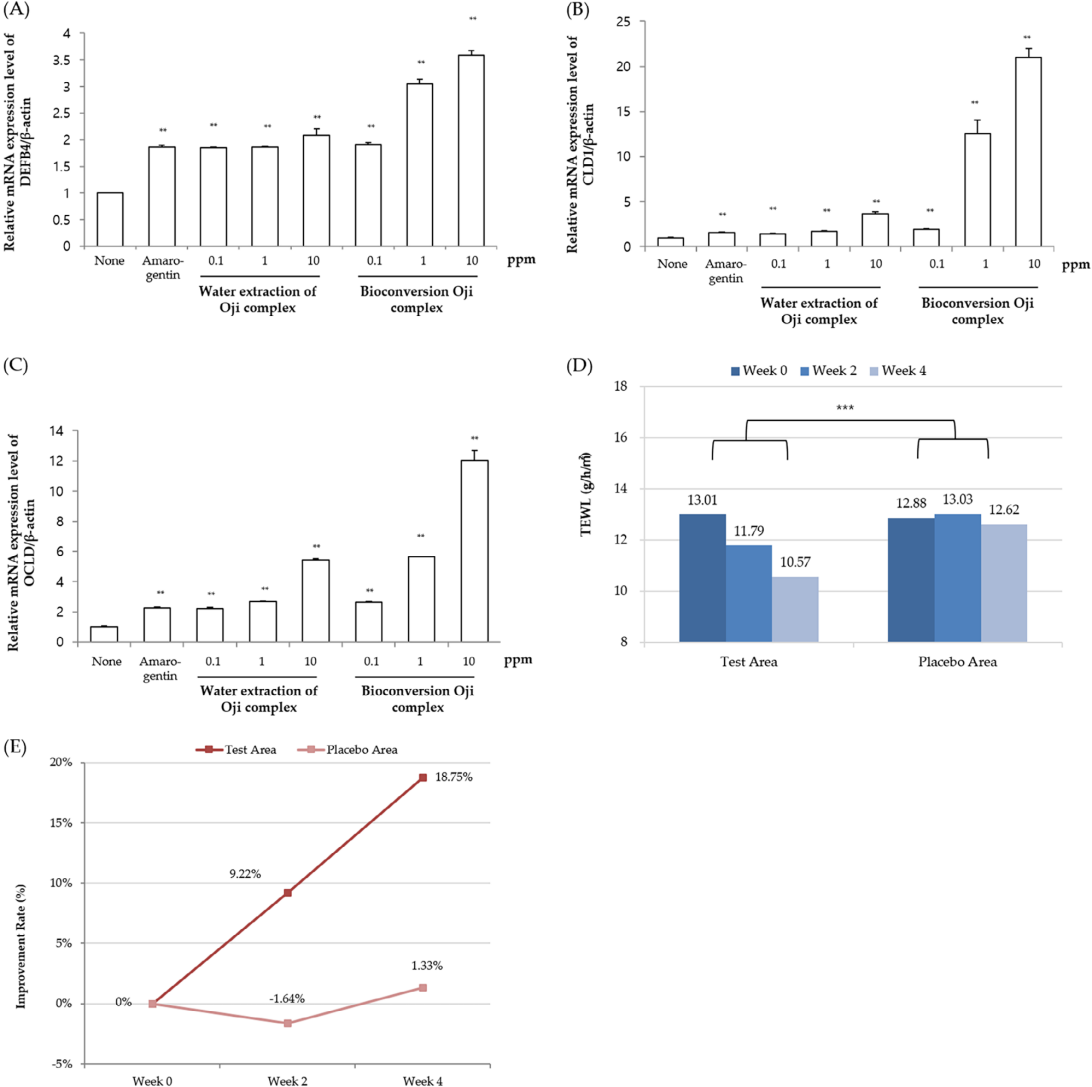
Skin barrier improving effects of Bioconversion Oji complex (Test sample) and water extraction of Oji complex (Placebo sample) on human skin in vitro and in vivo. The HaCaTs were treated with Bioconversion Oji complex or water extraction of Oji complex at the indicated doses for 24 h, and the relative mRNA levels of (A) DEFB4, (B) CLD1 and (C) OCLD were measured by qRT‐PCR. The means ± SEs are the average of three independent experiments. ^**^ *p* < 0.01, ^*^ *p* < 0.05 compared to the non‐treated group. In clinical study, (D) Changes of the skin water content and Transepidermal Water Loss (TEWL) and (E) Improvement rate of the TEWL were measured and calculated by Tewameter TM300 instrument. Data (*n* = 21) represent mean. ^***^ *p* < 0.001 indicated a statistically significant differences compared to non‐treated control, ^##^ *p* < 0.01, ^###^ *p* < 0.001 indicates a statistically significant differences compared to placebo control.

Beta‐defensin is a polypeptide composed of less than 100 amino acids found in host defense settings, which has an antibactierial activity in physiological concentration.^41^ It is known that beta‐defensin not only has an antibacterial effect on the epidermis, but also strengthens the tight junction between epidermal cells by increasing the expression of the Claudin family.^42^ Beta‐defensin is mainly present in the epidermal layer,^43^ and it has been reported that HaCaT used in this experiment expresses beta‐defensin gene (DEFB4).^44^ The substance used as a positive control for inducing beta defensin is amarogentin, which has been reported to increase beta defensin.^45^ Amarogentin mainly plays a role in regulating immunity between the epidermis and immune cells, and in particular, the effect of alleviating IL‐8, and inflammatory cytokine induced by histamine and TNF‐alpha, is already known.^46^ In addition, when epidermal cells were treated with amarogentin, the expression of skin barrier related genes such as Keratin 10 and Involucrin increased, proving the correlation between skin immunity and skin barrier.^46^ Therefore, we examined how the expression of beta‐defensin and skin barrier‐related genes are regulated to confirm the immunity enhancement and barrier improvement efficacy of Bioconversion Oji complex. As a result of the experiment, mRNA expression level of beta‐defensin was increased by Amarogentin, and the effect of increasing beta‐defensin expression was confirmed in Bioconversion Oji complex and Water extraction of Oji complex. In particular, the efficacy of increasing in bet‐defensin by about 1.75 times in Bioconversion Oji complex compared to the Water extraction of Oji complex was shown (Figure 4A). Also, the effect of increasing the expression of CLD1 (claudin 1) and OCLD (occludin) of the claudin family, which is known to be increased by beta defensin, was confirmed. In particular, it was observed that CLD1 increased by about 5 times or more, and OCLD increased by about 2 times or more (Figure 4B,C ). In clinical study, testing on 21 subjects during the 4 weeks test period, the test area using the Bioconversion Oji complex sample showed a statistically significant (*p* < 0.05) decrease in the TEWL after 2 weeks of sample application compared to Water extraction of Oji complex for placebo sample application. Also, a statistically significant difference (*p* < 0.05) was shown in comparison with the placebo area with Water extraction of Oji complex for placebo sample (Figure 4D,E).

## 4 DISCUSSION

Recently, plants used as the medicinal herbs in the folk remedy have been analyzed and evaluated for their skin efficacy with more concrete scientific data. Based on the *Journal of Hosancheong* and Limsanyeojibub, we have selected the five different plants such as *Prunus mume*, *Prunus persica*, *Morus alba*, *Salix alba*, and *Sophora japonica*, and applied bio conversion extraction method to improve the bioactivity of plant complexes. Microbially produced biosurfactants, such as *Candida bombicola*’s sophorolipids, are gaining popularity due to their biodegradability, low toxicity, and skin‐friendly properties, making them ideal for use in cosmetics.^11^, ^12^ In this study, macadamia seed oil was used as a source to produce biosurfactants from *Candida bombicola* for the first step of main fermentation. Five natural plants as mentioned above (*Prunus mume*, *Prunus persica* (Peach), *Morus alba*, *Salix alba* (Willow), *Sophora japonica*) were subsequently added to carry out the second step of main fermentation. Through the application of bioconversion technology, we observed the increased content of amino acids, as well as amphiphilic lipids that are renowned to be the components of human skin within Bioconversion Oji complex compared to Water extraction of Oji complex. Additionally, we identified 23 flavonoids within Bioconversion Oji complex. Many studies have already shown that those biologically active compounds including amino acid, polyphenols and lipids contribute to the skin moisturization and barrier function improvement as well as skin anti‐inflammatory effect.^47^

Based on the oriental medicine origin of Ojitang, we conducted a literature review on the vulnerability of baby skin. Baby skin has a thin and weak skin barrier, so there is relatively small amount of NMFs present,^48^ having thinner epidermal layer, and higher pH property compared to adult skin.^49^ Besides baby skin is susceptible to lose more moisture than the adult one because of its weak skin barrier; however, it shows high moisture absorption into skin.^49^ Therefore, in vitro and in vivo experiments were conducted focusing on the actives produced during bio conversion process. It markedly increased the amount of water retained by increasing NMFs that attracts and bind atmospheric water as well as internal water supplied from the dermis and has the effect of forming a strong skin barrier.

In this work, the toxicity of the Bioconversion Oji Complex was evaluated on HaCaT cell line. Subsequently, several tests performed to identify various biomarkers related to anti‐inflammation, skin moisture, and skin barrier in HaCaTs with Bioconversion Oji complex at a concentration confirmed to be non‐toxic. The Bioconversion Oji complex treatment caused significant effects in all parameters. The Bioconversion Oji complex was applied in three different concentrations, while the water extraction of Oji complex was used at the same concentration. The decreases of the inflammatory cytokine genes were found to be dose dependent in Bioconversion Oji complex. Also, the biomarkers related to skin hydration, skin barrier improvement and skin protection increase in dose dependent manners in Bioconversion Oji complex treated HaCaTs. Also, when compared to Water extraction of Oji complex, Bioconversion Oji complex shown significant effects on skin relief, skin hydration and barrier improvement in clinical studies. Therefore, the Bioconversion Oji complex exhibits significantly superior beneficial effects on the skin compared to Water extraction of Oji complex counterpart., which can be inferred due to various kinds and higher content of amino acids, lipids, and flavonoids through bioconversion technology grafting.

## 5 CONCLUSIONS

Bioconversion technology is increasingly welcomed in natural product research area because of its various features, such as such as biodegradability, biocompatibility and physicochemical, which are far better than the traditional chemical synthesis and natural medicine separation technology. Oil bioconversion technology *using Candida bombicola* was newly applied to maximize the extraction efficiency and efficacy of amino acids and the content of new bioactive compounds like lipids and flavonoids in Ojitang. Due to their biosurfactant producing capabilities, Bioconversion Oji complex demonstrates a significant capacity for enhancing skin hydration, protection of the skin barrier and alleviating skin irritation.

## CONFLICT OF INTEREST STATEMENT

The authors declare no conflict of interest.

## INSTITUTIONAL REVIEW BOARD STATEMENT

The study was conducted according to the guidelines of the Declaration of Helsinki, and approved by the Institutional Review Board (GMRC, IRB no. KDRI‐IRB‐21962, KDRI‐IRB‐21963, KDRI‐IRB‐21964) for studies involving humans.

## INFORMED CONSENT STATEMENT

Informed consent was obtained from all subjects involved in the study. Written informed consent has been obtained from the patients to publish this paper.

## Supporting information


Supporting Information

## Data Availability

The data are available from the corresponding author upon reasonable request.
