# Supplementary material for: A novel sophorolipids extraction method by yeast fermentation process for enhanced skin efficacy
Source: Skin Res Technol. 2023 Nov 13;29(11):e13518. doi: 10.1111/srt.13518 (PMC10643984; doi:10.1111/srt.13518)
Supplement: Supplementary file 1 — Supporting Information [file SRT-29-e13518-s001.docx]

**Supplementary Materials:** Table S1: Operating condition of HPLC for analyzing lipids in Water extraction of Oji complex and Bioconversion Oji complex, Table S2: Operating condition of TOF/MS for analyzing lipids in Water extraction of Oji complex and Bioconversion Oji complex, Table S3: Operating condition of HPLC for analyzing flavonoids in Bioconversion Oji complex, Table S4: Operating condition of TOF/MS for analyzing flavonoids in Bioconversion Oji complex, Figure S1: HPLC chromatogram of free amino acids in Water extraction of Oji complex, Figure S2: HPLC chromatogram of free amino acids in Water extraction of Oji complex, Figure S3: HPLC chromatogram of flavonoids in Bioconversion Oji complex., Figure S4: HPLC chromatogram of lipids in Bioconversion Oji complex.

**Author Contributions:** Y.L and Y.C contributed to the experiment design, performed the data, collected the data, and wrote the manuscript. S. J., S. Y.,Y.N and S.h.K contributed to manuscript writing, revising it. W.K helped with data, provided clinical data. J. S, and J. K helped with data, provided material analysis data. S.y.K provided experimental support and supervision. All authors have read and agreed to the published version of the manuscript.

**Funding:** This research received no external funding

**Institutional Review Board Statement:** The study was conducted according to the guidelines of the Declaration of Helsinki, and approved by the Institutional Review Board (GMRC, IRB no. KDRI-IRB-21962, KDRI-IRB-21963, KDRI-IRB-21964) for studies involving humans.

**Informed Consent Statement:** Informed consent was obtained from all subjects involved in the study. Written informed consent has been obtained from the patients to publish this paper.

**Data Availability Statement:** The data are available from the corresponding author upon reasonable request.

**Conflicts of Interest:** The authors declare no conflict of interest.

Supplementary data

**Table S1.** Operating condition of HPLC for analyzing lipids in Water extraction of Oji complex and Bioconversion Oji complex

| **Operation conditions of HPLC** | | |
| --- | --- | --- |
| **Column** | Waters BEH C18 (2.1 mm X 100 mm, 1.7 um) | |
| **Flow rate**  **(mL/min)** | 0.4 | |
| **Column temp (°C)** | 45 °C | |
| **Solvent (%)** | A: 0.1% acetic acid, 10mM ammonium formate in acetonitrile /water (6:4)  B: 0.1% acetic acid, 10mM ammonium formate in acetonitrile /Isopropyl alcohol (1:9) | |
| **Time (min)** | **A (%)** | **B (%)** |
| 0 | 95 | 5 |
| 0.5 | 95 | 5 |
| 20 | 0 | 100 |
| 25 | 0 | 100 |
| 25.5 | 95 | 5 |
| 30 | 95 | 5 |

**Table S2.** Operating condition of TOF/MS for analyzing lipids in Water extraction of Oji complex and Bioconversion Oji complex

| **Ionization source** | Electrospray ionization (ESI) |
| --- | --- |
| **Ionization mode** | Positive and negative |
| **MS scan type** | Full scan and Information Dependent Acquisition (IDA) Scanning |
| **MS scan range** | 100 ~ 2000 m/z |
| **MS /MS scan range** | 30 ~ 2000 m/z |
| **Gas Temperature** | 500 ℃ |
| **Ionspray voltage** | Positive: 5.5kV, Negative: 4.5 kV |
| **Nebulizing gas** | 50 psi |
| **Heating gas** | 50 psi |
| **Collision gas** | N₂ |
| **Collision Energy** | POS: 35±15, Neg: -35±15 |
| **Collision Energy (CE)** | POS: 10, Neg: -10 |
| **Declustering Potential (DP)** | POS: 60, Neg: -60 |

**Table S3.** Operating condition of HPLC for analyzing flavonoids in Bioconversion Oji complex

| **Operation conditions of HPLC** | | |
| --- | --- | --- |
| **Column** | Waters Cortex C18 (2.1 mm X 150 mm, 1.6 µm) | |
| **Flow rate (mL/min)** | 0.4 | |
| **Column temp (°C)** | 45 °C | |
| **Solvent (%)** | A: 0.1% formic acid in water  B: 0.1% formic acid in acetonitrile | |
| **Time (min)** | **A (%)** | **B (%)** |
| 0 | 99 | 1 |
| 1 | 99 | 1 |
| 13 | 75 | 25 |
| 21 | 0 | 100 |
| 24 | 0 | 100 |
| 26 | 99 | 1 |
| 30 | 99 | 1 |

**Table S4.** Operating condition of TOF/MS for analyzing flavonoids in Bioconversion Oji complex

| **Ionization source** | Electrospray ionization (ESI) |
| --- | --- |
| **Ionization mode** | Positive and negative |
| **MS scan type** | Full scan and Information Dependent Acquisition (IDA) Scanning |
| **MS scan range** | 50 ~ 2000 m/z |
| **MS /MS scan range** | 50 ~ 2000 m/z |
| **Gas Temperature** | 500 ℃ |
| **Ionspray voltage** | Positive: 5.5kV, Negative: 4.5 kV |
| **Nebulizing gas** | 50 psi |
| **Heating gas** | 50 psi |
| **Collision gas** | N₂ |
| **Collision Energy** | POS: 35±15, Neg: -35±15 |
| **Collision Energy (CE)** | POS: 10, Neg: -10 |
| **Declustering Potential (DP)** | POS: 60, Neg: -60 |


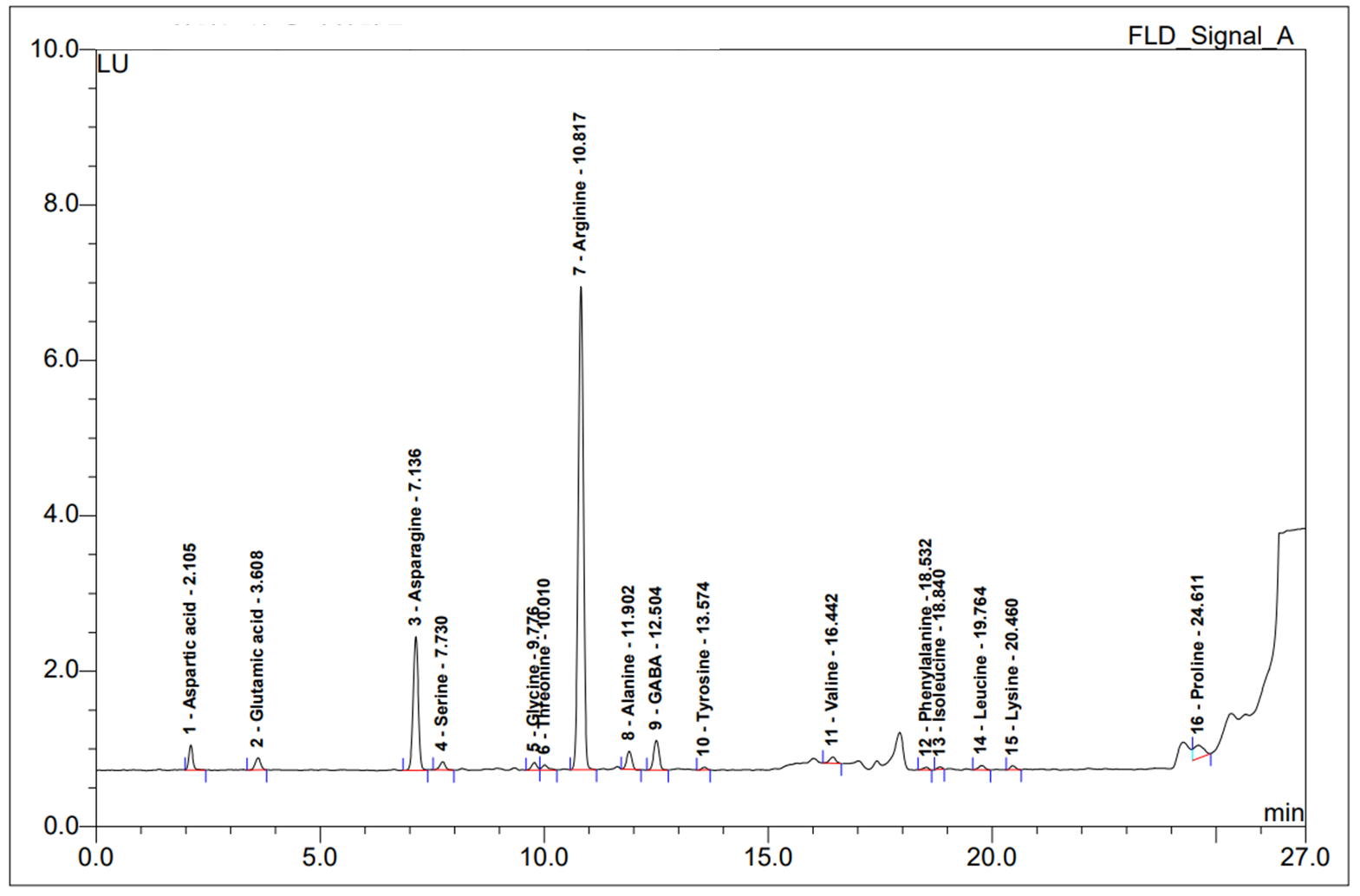


**Figure S1**. HPLC chromatogram of free amino acids in Water extraction of Oji complex


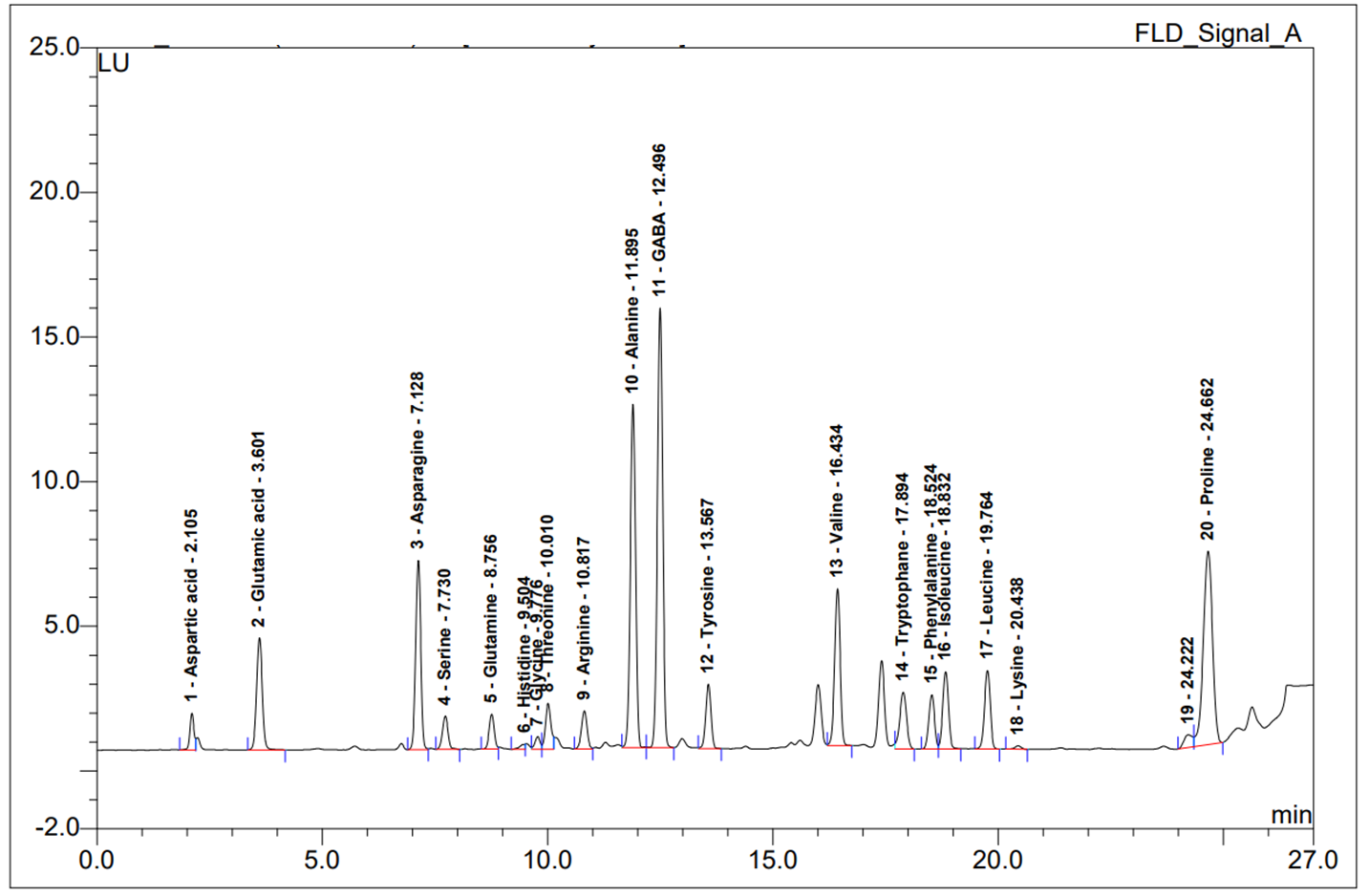


**Figure S2**. HPLC chromatogram of free amino acids in Water extraction of Oji complex


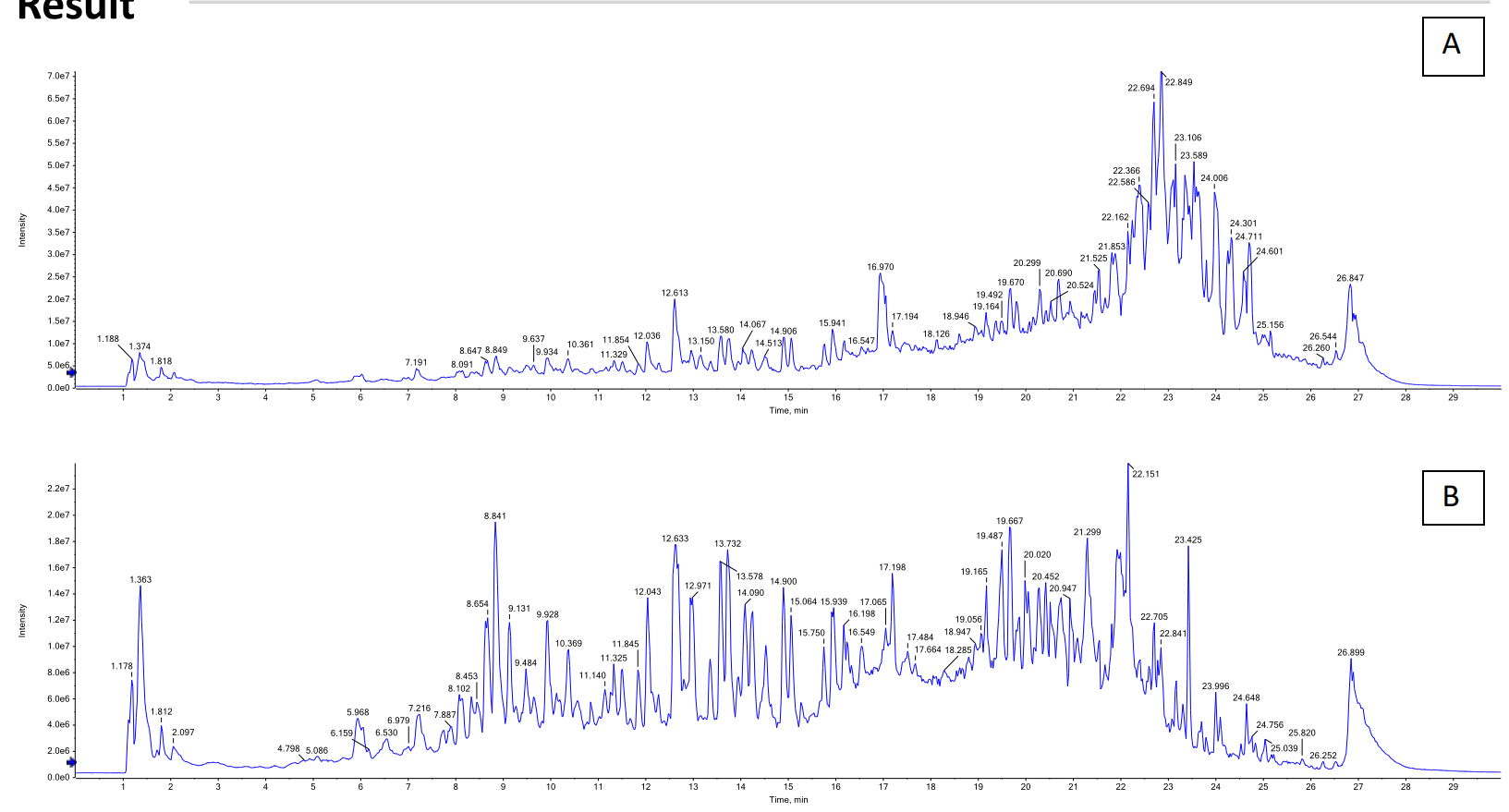


**Figure S3**. HPLC chromatogram of flavonoids in Bioconversion Oji complex. HPLC chromatogram (210 nm) and TOF-MS TIC Positive (A) and HPLC Chromatogram (210nm) and TOF-MS TIC Negative (B)


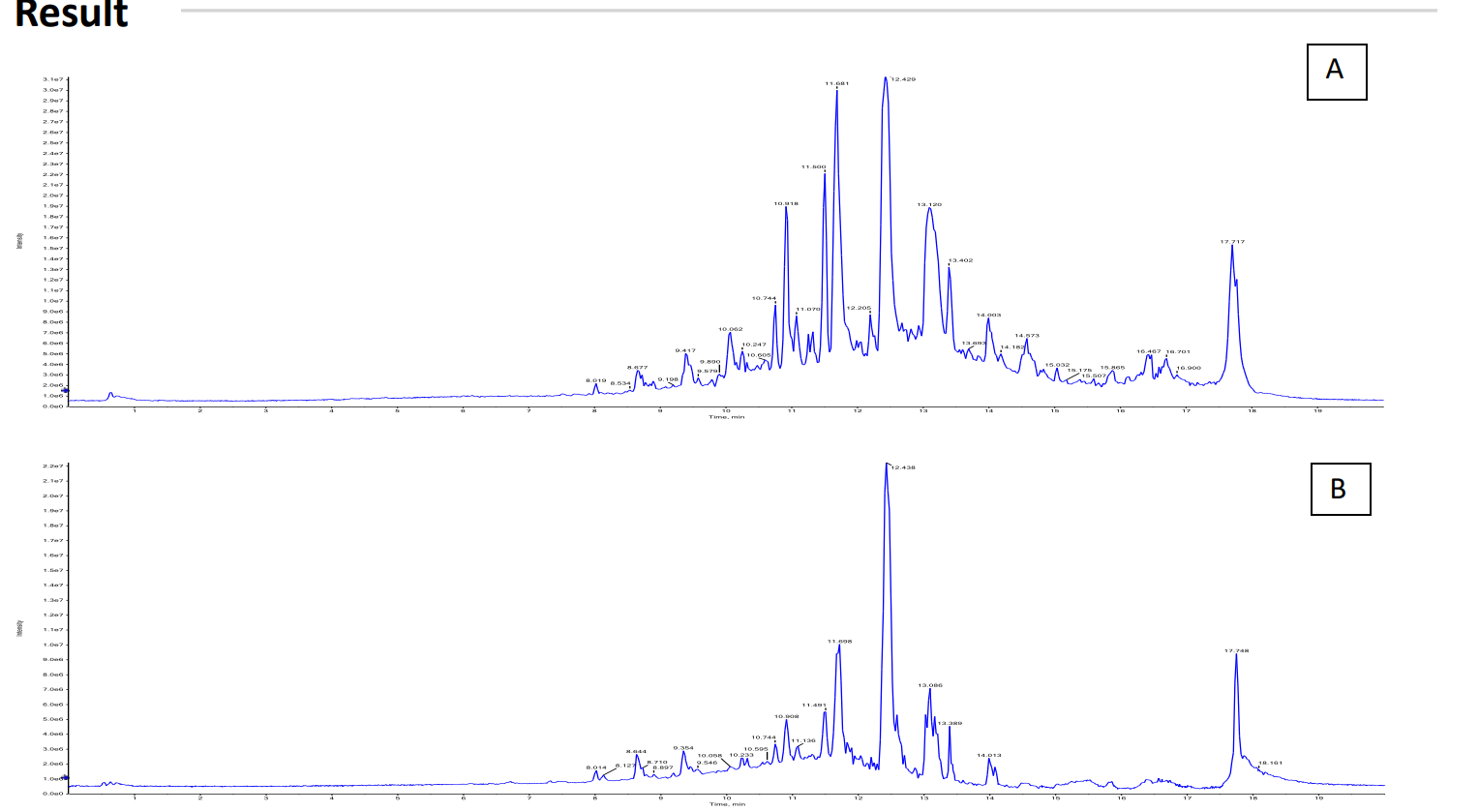


**Figure S4**. HPLC chromatogram of lipids in Bioconversion Oji complex. HPLC chromatogram (210 nm) and TOF-MS TIC Positive (A) and HPLC Chromatogram (210nm) and TOF-MS TIC Negative (B)
